# Editorial: Novel biomarkers and extracellular vesicles in endocrine hypertension and related disorders

**DOI:** 10.3389/fendo.2023.1262046

**Published:** 2023-08-14

**Authors:** Cristian A. Carvajal, Morag J. Young, Damian G. Romero

**Affiliations:** ^1^ Department of Endocrinology, School of Medicine, Pontificia Universidad Católica de Chile, Santiago, Chile; ^2^ Center for Translational Research in Endocrinology (CETREN-UC), Pontificia Universidad Católica de Chile, Santiago, Chile; ^3^ Cardiovascular Endocrinology Laboratory, Baker Heart and Diabetes Institute, Melbourne, VIC, Australia; ^4^ Baker Department of Cardiometabolic Health, University of Melbourne, Melbourne, VIC, Australia; ^5^ Department of Cell and Molecular Biology, University of Mississippi Medical Center, Jackson, MS, United States; ^6^ Mississippi Center of Excellence in Perinatal Research, University of Mississippi Medical Center, Jackson, MS, United States; ^7^ Women’s Health Research Center, University of Mississippi Medical Center, Jackson, MS, United States; ^8^ Cardiovascular-Renal Research Center, University of Mississippi Medical Center, Jackson, MS, United States

**Keywords:** extracellular vesicles (EVs), endocrine hypertension, renal disease, biomarkers, exosome

The search for novel or complementary biomarkers, including extracellular vesicles (EVs) or exosomes, is a highly active research field (1). Biomarkers, EVs and EV-cargos have been suggested to be highly useful for patient’s stratification, diagnosis, and prognosis for endocrine hypertension, and also for cardiovascular and renal disorders (1, 2).

EVs have been highlighted for their potential as a form of liquid biopsy that contains multiple types of “cargo” including cell-specific miRNA species and proteins. EVs have enormous potential for identification of novel metabolic and pathophysiological mechanisms affecting cardiovascular health, biomarker discovery and to advance in development of novel therapeutic strategies. Urinary exosomes are of particular interest since they can be isolated from urine in a non-invasive fashion and from large volumes of low concentration samples (3). Both standard analytical methods and high-throughput omics technologies have been applied in (urinary) EV biomarker research (4), leading to the identification of novel regulatory pathways and also the discovery of several potential EV-based biomarkers for a range of diseases, including cancer and chronic diseases (5).

This Research Topic covers recent research in urinary EVs and novel biomarkers associated to endocrine hypertension, primary aldosteronism (PA) and renal diseases (2). It consists of two reviews, two original research manuscripts and a perspective.

The review by Wu et al. “*Using human urinary extracellular vesicles to study physiological and pathophysiological states and regulation of the sodium chloride cotransporter*” provides a brief overview of the state-of-the-art, challenges and knowledge gaps in current uEV-based analyses, with a focus on the application of uEVs to study the “renal-K^+^ switch” and the thiazide-sensitive sodium chloride cotransporter (NCC) regulation. They also provide recommendations regarding biospecimen handling, processing, and reporting requirements to improve experimental reproducibility of studies associated to discover uEV-derived biomarkers in arterial hypertension and clinical practice. The authors reported the use of uEVs as a tool to assess NCC abundance and activity in a variety of human studies has provided insight to the relative roles of K^+^ and mineralocorticoids in NCC regulation and its potential pitfalls associated to age, sex, and states of disease.

In the review by Araos and Amador “*Neutrophil Gelatinase Associated- Lipocalin as an Immunomodulator in Endocrine Hypertension*” the link between neutrophil gelatinase-associated lipocalin (NGAL) and endocrine hypertension is discussed with a focus on PA. Possible regulators and mechanisms are discussed in detail with particular attention paid to the role of NGAL as an immunomodulator. In the last decade, studies have shown that NGAL is required to develop aldosterone-induced hypertension and is associated with end-organ damage (6). NGAL has multiple origins, from epithelial cells to immune cells, is modulated by microRNAs and transported by extracellular vesicles which support its use as a biomarker for endocrine hypertension due to increased circulating aldosterone (7). The authors reviewed in detail the supportive evidence arising from recent studies in the area that support a role for MR-dependent activation in antigen-presenting cells (APCs) and induction of NGAL. APC-derived NGAL promotes differentiation and recruitment of Th lymphocytes, through the release of specific mediators indicating a pathway for MR regulation of Th lymphocytes (6). Given that NGAL has been found in EVs, it adds a novel route of communication and mechanism of this lipocalin in its role as pro-inflammatory and pro-fibrotic mediator, either *via* MR or independent of MR, highlighting its role in endocrine hypertension morbidity.

The original manuscript from Bertolone et al. “*Proteomic analysis of urinary extracellular vesicles highlights specific signatures for patients with Primary Aldosteronism*”, showed that UEVs are molecular biomarkers associated to PA characterization highlighting decreased expression of AQP1 and AQP2 in PA compared with essential hypertension (EH). They studied the proteome of UEVs from patients with PA and essential hypertension EH patients, and identified specific molecular indicators associated with pathophysiological features of PA, including those related to either aldosterone producing adenoma (APA) or bilateral primary aldosteronism (BPA). Using proteomic analysis of UEVs the authors identified differences in water reabsorption proteins such as AQP1 and AQP2 in PA, and specific proteomic signatures in APA vs BPA.

The original manuscript by Carotti et al. “*Involvement of ceramide biosynthesis in increased extracellular vesicle release in Pkd1 knock out cells*” they found a role of EVs associated to Autosomal Dominant Polycystic Kidney Disease (ADPKD) that is an inherited disorder characterized by the development of renal cysts, which frequently leads to renal failure. Using Pkd1 deficient mouse cells of both the distal convoluted tubule (DCT) and inner medullary collecting duct (IMCD), the authors demonstrated a significant increase in EV release in Pkd1^-/-^ mDCT15 and mIMCD3 cells, with respect to the wild type cells. Elevated EV release was associated with changes in the purinergic signaling and ceramide biosynthesis enzymes, suggesting the involvement of the DCT in the EV-mediated ADPKD progression and points to the induction of ceramide biosynthesis as an underlying molecular mechanism. These findings highlight the role of purinergic activity and the biosynthesis of ceramide in EVs generation in TCD and CD, which have the potential to promote renal failure and further arterial hypertension and other cardiovascular symptoms observed in patients with kidney alterations.

Finally, the perspective from Friso et al. “*Urinary extracellular vesicles carry valuable hints through mRNA for the understanding of endocrine hypertension*” enlighten the reader about the state-of-the-art and the possible future use of uEVs transcriptomics to build our understanding of the pathophysiology of hypertension, as well as, diagnostic-prognostic approaches. UEVs are now well recognized as a valuable source of information about the originating tissues, that can be obtained through a readily available non-invasive procedure. Urinary EVs can carry protein and nucleic acids, especially RNA, and therefore represent a unique way to perform gene expression analysis of a tissue strictly related to the pathophysiology of arterial hypertension such as the kidney (3). In this perspective, Friso et al. highlight to advance uEVs transcriptomics and proteomics to reach a deeper knowledge on the physiology and pathophysiology of arterial hypertension, especially mineralocorticoid-dependent hypertension, than eventually would lead to the design of more specific diagnostic-prognostic strategies.

In summary, this Research Topic is focused on advances in EVs, particularly uEVs, in humas and mice models associated to endocrine hypertension and kidney pathology. The series of manuscripts on this topic emphasize uEVs as useful biological tool to reveal proteins or molecular signatures related to specific disease etiologies. The EV-cargo reflects the molecular content of the parent cells from which they are released and thus carry cell specific markers from every segment of the nephron and urogenital tract (8). Paired with the non-invasive collection of a large quantity of sample exosomes, are thus ideal for evaluating the health status of these systems, and have great potential as multiplex-biomarkers associated with key subtypes of hypertension and renal disease (3, 8) (**Figure 1**). Further studies investigating the regulatory mechanisms associated to EV release, EV-cargo and paracrine or endocrine cell-cell communication are warranted to advance in the use of uEVs in the diagnosis, prognosis and potential treatment of different diseases affecting the cardiovascular system as well as many renal disorders.

**Figure 1:**
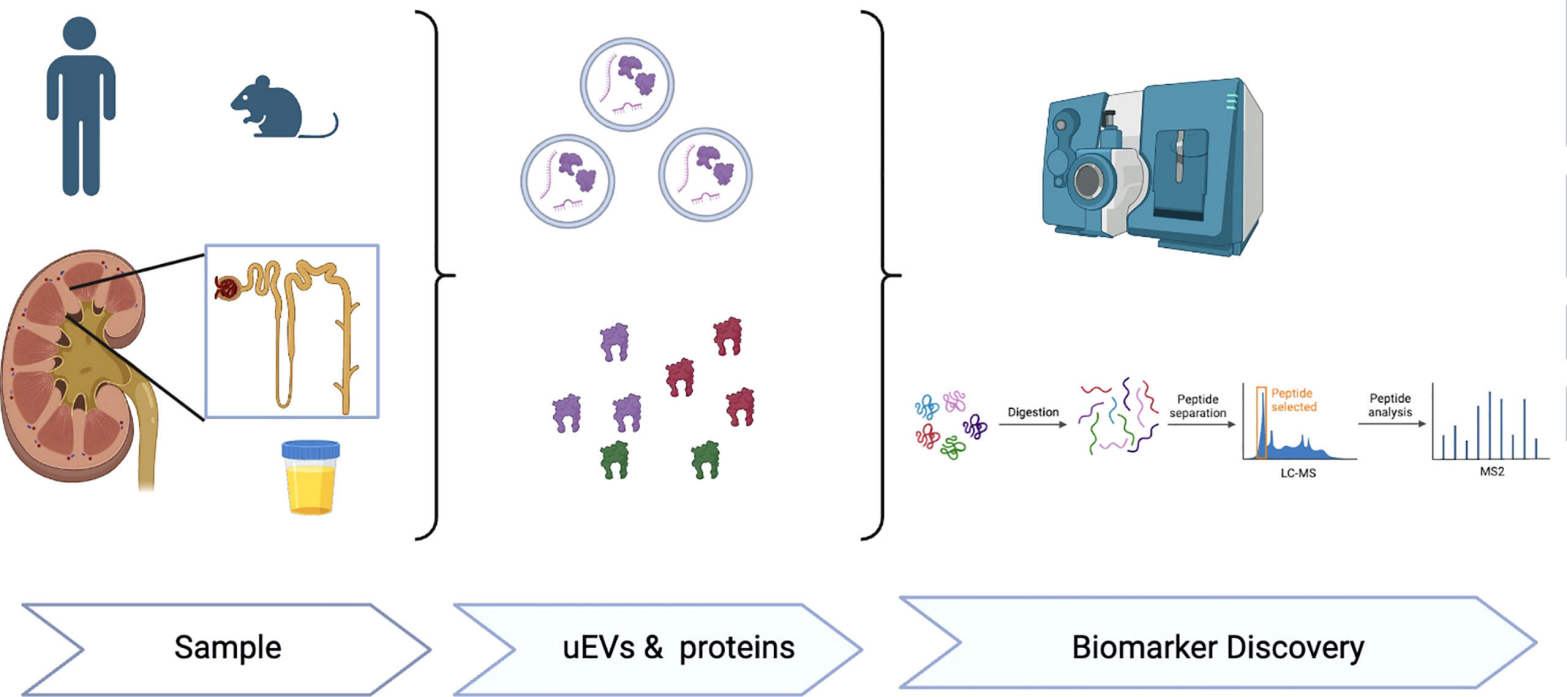
Extracellular vesicles (EVs), either plasma or urinary EVs are a novel biological tool to identify proteins or EV molecular signatures (RNA, miRNA, proteins) useful for novel research and biomarker discovery associated to hypertension, cardiovascular and renal diseases.

## Author contributions

CC: Conceptualization, Funding acquisition, Investigation, Writing – original draft, Writing – review & editing. MY: Writing – original draft, Writing – review & editing. DR: Writing – original draft, Writing – review & editing.
